# The profile of polyunsaturated fatty acids in juvenile idiopathic arthritis and association with disease activity

**DOI:** 10.1007/s10067-017-3586-9

**Published:** 2017-02-28

**Authors:** Daiva Gorczyca, Jacek Postępski, Aleksandra Czajkowska, Mariola Paściak, Anna Prescha, Edyta Olesińska, Anna Gruenpeter, Iwona Lachór-Motyka, Bogumiła Szponar

**Affiliations:** 1 Third Department and Clinic of Paediatrics, Immunology and Rheumatology of Developmental Age, Wroclaw Medical University, Wroclaw, Poland; 2 First Department of Paediatrics Pulmonology and Rheumatology, University of Medicine in Lublin, Lublin, Poland; 3 Hirszfeld Institute of Immunology and Experimental Therapy, Polish Academy of Sciences, Wroclaw, Poland; 4 Department of Food Science and Dietetics, Wroclaw Medical University, Wroclaw, Poland; 5 Department of Paediatric Rheumatology, John Paul II Paediatric Centre, Sosnowiec, Poland

**Keywords:** Dietary intake, Disease activity, Inflammatory markers, Juvenile idiopathic arthritis, n-3 PUFA, n-6 PUFA

## Abstract

We investigated the association between dietary intake of n-3 and n-6 polyunsaturated fatty acids (PUFAs), serum profiles, and immune and inflammatory markers in juvenile idiopathic arthritis (JIA) in relation to onset, activity, and duration. A total of 66 JIA patients and 42 controls were included. Serum PUFA levels were assessed by gas-liquid chromatography-mass spectrometry, a dietary intake by 7-day dietary record method, and IL-6, IL-10, and IL-17A levels using ELISA. Dietary PUFA intake did not differ between the JIA group and controls. Intakes of n-6 and n-3 PUFA and serum levels were not associated. Levels of total n-6 PUFA and linoleic acid (LA) were higher in inactive JIA than in active JIA. Patients with active and short-lasting disease (less than 3 months from diagnosis) had significantly lower levels of arachidonic acid (AA) and docosahexaenoic acid (DHA) than the control. Serum α-linolenic acid (ALA) levels were significantly higher in poly-JIA than in oligo-JIA and in controls. We found significantly higher serum IL-10 levels in JIA than in controls. Serum n-6 and n-3 levels were significantly negatively correlated with active joint count, erythrocyte sedimentation rate, and C-reactive protein and positively with platelet count. Our study presents the low levels of AA and DHA in the active phase of short-lasting JIA, particularly poly-JIA, and the relationship between n-6 and n-3 PUFA and classic markers of inflammation. PUFAs may contribute to the pathogenesis of JIA and support a necessity to identify new targets suitable for successful interventional studies in JIA patients.

## Introduction

Juvenile idiopathic arthritis (JIA) is the most common chronic inflammatory articular disease in childhood. Elevation of pro-inflammatory cytokines has been demonstrated in both serum and synovial fluid of patients with JIA: tumor necrosis factor-α (TNF-α), interferon-γ (IFN-γ), interleukin-6 (IL-6), and interleukin-1β (IL-1β), usually in the active phase of the disease [1–3]. In remission, the anti-inflammatory interleukin-10 (IL-10) produced by T-regulatory cells predominates [4].

Fatty acids have numerous functions within the body: they are structural components of cell membranes, an important source of energy, and act as signaling molecules. Among them are long-chain polyunsaturated fatty acids (PUFAs), precursors of endogenous synthesis of pro-inflammatory or anti-inflammatory lipid mediators. n-6 (omega-6) and n-3 (omega-3) series of PUFA are metabolized by the same set of enzymes. The membranes of monocytes and macrophages typically contain large amounts of arachidonic acid (20:4n-6, AA) compared to a minute load of docosahexaenoic acid (22:6n-3; DHA) and eicosapentaenoic acid (20:5n-3, EPA). However, the role of EPA and DHA in inhibition of T cell proliferation, decrease in the chemotactic response of leukocytes, reduction of adhesion molecule expression, and decrease in the production of inflammatory cytokines requires further explanation [5].

The arachidonic acid cascade has become a starting point for a dietary approach based on n-3 PUFA supplementation and dietary modification of patients with rheumatoid arthritis (RA). The concern is based primarily on the contention that a diet rich in linoleic acid (18:2n-6, LA) would promote tissue AA accumulation, enhance the production of pro-inflammatory eicosanoids derived from AA, and/or inhibit the conversion of α-linolenic acid (18:3n-3, ALA) to EPA and DHA and their subsequent metabolism to predominantly anti-inflammatory compounds [6, 7]. Some studies indicated that manipulation in dietary fatty acids can reduce a number of swollen and tender joints in patients with RA [8, 9], but others do not confirm these findings [10].

Few records were published regarding the role of PUFA in JIA. Gheita et al. [11] demonstrated the effect of n-3 PUFA supplementation on laboratory and clinical parameters of inflammation. Górska et al. [12] determined the levels of PUFAs in relation to cytokines in the active phase of the JIA.

Several studies on the direct relationship between dietary intake of PUFA and their level in cell membrane phospholipids, adipose tissue, serum, or plasma in a healthy adult population were published [13–15], but the findings in the pediatric population are different [16, 17]. Moreover, there is a shortage of studies comparing the dietary intake of PUFA and serum PUFA level in patients suffering from different chronic inflammatory diseases, with JIA in this number.

We performed this study with the aim of finding the association between dietary intake of n-3 and n-6 PUFA, serum PUFA profile, and immune and inflammatory markers. We hypothesized that disturbances of the PUFA metabolism pathway are dependent on JIA subtype, disease activity, and disease duration.

## Subjects and methods

A total of 66 children with JIA were enrolled in this study, from three pediatric rheumatology clinics in three centers in Poland: (i) the Clinic of Paediatrics Rheumatology in Wroclaw Medical University, (ii) the Department of Paediatric Pulmonology and Rheumatology Medical University of Lublin, and (iii) the Department of Children Rheumatology in John Paul II Paediatrics Centre in Sosnowiec, between October 2012 and December 2014. The diagnosis of JIA was based on International League of Associations for Rheumatology (ILAR) criteria [18].

The patients underwent clinical examination by a pediatric rheumatologist. The following data was collected: age at disease onset, age at consent, duration of disease, number of active/swollen joints, number of joints with limitation of motion, presence of uveitis, duration of morning stiffness, the presence of RF, anti-cyclic citrullinated peptide (anti-CCP) antibodies, anti-nuclear antibody (ANA) positivity, and treatment (drug and dosage).

The patients with JIA were categorized into six subgroups according to disease subtype, disease activity, and disease duration from diagnosis. We used the following criteria to define these groups. The JIA subgroups were defined upon ILAR criteria [18]. Clinical active JIA was defined as the presence of at least one joint swelling or limitation of joint motion in association with pain, warmth, or tenderness of the joint. Clinical inactive JIA was defined as no joints with active arthritis, no fever, rash, serositis, splenomegaly, or generalized lymphadenopathy attributable to JIA, no active uveitis, erythrocyte sedimentation rate (ESR) or/and C-reactive protein (CRP) level within normal limits, and duration of morning stiffness ≤15 min. Short-lasting disease was defined as disease duration less than 3 months from diagnosis and long-lasting disease as more than 3 months from diagnosis.

Healthy children were enrolled in the study as a control group. They had outpatient follow-up in the hospital for routine medical check-up.

The exclusion criteria for participants of both groups were chronic autoimmune diseases other than JIA, gastrointestinal diseases (inflammatory bowel disease, gastritis, and duodenal ulcers), acute febrile illness, patients on biological therapy, taking supplements containing n-6 and/or n-3 PUFAs, and antioxidants.

The study protocol was approved by the Ethics Committee of Wroclaw Medical University (No. KB 390/2012).

Children’s parents or legal representatives signed a written informed consent to participate in the study.

## Methods

### Anthropometric measurements

The body weight and height of all children were measured and BMI was calculated. Current recommendations for the evaluation of nutritional status of children in a clinical setting regarding body weight, height, and BMI percentiles have been the most useful approach [19]. Our records of the anthropometric measurements were calculated using current Polish growth charts, followed by height, weight, and BMI parameters in comparison to the relevant percentile curves [20].

### The dietary PUFA intake

The analysis of the dietary intake by each study participant was carried out using a 7-day dietary recording method. In order to minimize the influence of season on nutrient intake, the number of participants in each studied group was matched with a season of the year. The children’s guardians participated in the dietary recording. Data on food and beverages consumed and supplements taken during a 7-day period (a week) were collected and then verified by trained interviewers. The “Photo Album of Products and Meals” was used to estimate food portion sizes [21]. Data on physical activity (excluding children less than 7 years old) and taking dietary supplements were also collected. The daily energy values and an intake of food components including fat and fatty acids as well as participation of energy supply from macronutrients in individuals and studied groups were calculated using Dieta 5.0 software with Polish database (National Food and Nutrition Institute 2015, Warsaw, Poland).

### Blood samples and blood tests

On the day of medical examination, the venous blood samples were collected once from overnight fasted participants from the JIA group and the healthy control group. Serum was separated immediately and stored at −70 °C until the analysis of PUFAs and cytokines.

Complete blood count (CBC), ESR, and CRP, as inflammatory markers, were measured as part of routine clinical assessment in JIA patients.

### Polyunsaturated n-6 and n-3 fatty acid serum profiles

Serum n-6 PUFAs LA and AA and n-3 PUFAs ALA, EPA, and DHA were detected. The method for extraction and derivation of fatty acids was adapted from Böcking et al. [22]. The fatty acid methyl esters were analyzed using a gas chromatography-mass spectrometry (GLC-MS) system: Focus GC apparatus with ion trap ITQ 700 (Thermo) and Trace GC with TSQ Quantum XLS triple quadrupole (Thermo). For quantitative analysis, tricosanoic acid methyl ester (C23:0) was added to the examined samples as internal standard.

### Immune markers

The levels of IL-6 and IL-10 (BD Pharmingen, San Diego, CA) and IL-17A (eBiosciences, San Diego, CA) were determined in serum samples with enzyme-linked immunosorbent assays (ELISAs).

### Statistical analysis

Chi-squared tests (Pearson’s *χ* ^2^ test) were used for the background analysis to test for differences in qualitative variables between groups. Descriptive statistics were presented as median and range, respectively. Statistically significant differences between groups were analyzed using a Mann-Whitney test. Correlations between variable were examined using Spearman’s correlation coefficients. All probability values are two-tailed, and the level of significance was *p* < 0.05. Statistical analysis was performed using SPSS for Windows statistical software (SPSS Inc., Chicago, IL, USA).

## Results

The clinical characteristics of the participants in this study are summarized in Table 1. Both groups were similar according to age, sex, body height, weight, and BMI. All participants were Caucasian.

**Table 1:** Characteristics of participants

Parameter	JIA group, *n* = 66	Control group, *n* = 42	*p* value
Gender, *n* (%)			0.44
Male	16 (24.2)	13 (30.9)	
Female	50 (75.8)	29 (69.1)	
Age, years, median (range)	8.6 (1.5–18)	8.8 (4–17.8)	0.43
Body weight, kg, median (range)	25.05 (10.10–102.00)	28.95 (13.90–70.00)	0.26
Body weight, percentile, *n* (%)			
Less than 25	28 (42.4)	9 (21.4)	0.06
25–75	27 (40.9)	21 (50.0)	
Over 75	11 (16.7)	12 (28.6)	
Body height, cm, median (range)	127 (77–175)	128 (100–196)	0.38
Body height, percentile, *n* (%)			
Less than 25	20 (30.3)	5 (11.9)	0.07
25–75	35 (53.0)	30 (71.4)	
Over 75	11 (16.7)	7 (16.7)	
BMI, kg/m^2^, median (range)	16.45 (12.01–34.40)	17.05 (13.76–30.00)	0.46
BMI, percentile, *n* (%)			
Less than 25	24 (36.4)	10 (23.8)	0.36
25–75	27 (40.9)	19 (45.2)	
Over 75	15 (22.7)	13 (31.0)	
Subtype of JIA, *n* (%)			
Systemic arthritis	3 (4.5)		
Oligoarthritis	38 (57.8)		
Oligoarthritis persistent/excendent (*n*/*n*)	30/8		
Polyarthritis (RF-negative)	12 (18.2)		
Polyarthritis (RF-positive)	6 (9.1)		
Psoriatic arthritis	1 (1.5)		
Enthesitis-related arthritis	5 (7.6)		
Undifferentiated arthritis	1 (1.5)		
Age at disease onset, years, median (range)	6 (1.1–16)		
Disease duration, years, median (range)	1 (0.2–14)		
Active/non-active disease	53/13		
Joints with active disease, median (range)	2 (0–27)		
Duration of morning stiffness, min, median (range)	30 (0–120)		
Leucocytes/μL, median (range)	7.32 (2.35–62.60)		
Leucocytosis, *n* (%)	12 (18.2)		
Anemia, *n* (%)	5.00 (7.6)		
CRP level, mg/L, median (range)	0.88 (0.01–56.31)		
Elevated CRP level (>5 mg/L), *n* (%)	16 (24.2)		
ESR, mm/h, median (range)	15 (2–65)		
Elevated ESR (>20 mm/h), *n* (%)	27 (40.9)		
RF-positive, *n* (%)	12 (18.2)		
ANA-positive, *n* (%)	28 (42.4)		
Elevated anti-CCP level (>17 U/ml), *n* (%)	7 (10.6)		
Iridocyclitis, *n* (%)	5 (7.6)		
Treatment, *n* (%)			
Non-steroidal anti-inflammatory drugs	36 (54.5)		
Methotrexate	48 (72.7)		
Chloroquine or hydroxychloroquine	16 (24.2)		
Sulfasalazine	9 (13.6)		
Prednisolone	34 (51.5)		
No treatment	2 (3.0)		

*ANA* anti-nuclear antibodies, *anti-CCP* anti-citrullinated protein, *BMI* body mass index, *CRP* C-reactive protein, *ESR* erythrocyte sedimentation rate, *NSAID* non-steroidal anti-inflammatory drugs, *MTX* methotrexate, *PRE* prednisone, *RF* rheumatoid factor

Among the 66 patients with JIA, 38 had an oligo-articular JIA (oligo-JIA) and 18 had poly-articular JIA (poly-JIA) (RF-positive and RF-negative). Ten patients having less common subtypes (systemic JIA (sJIA), psoriatic arthritis, enthesitis-related arthritis, and undifferentiated arthritis) were combined into one group because of small numbers and were excluded from analysis. Fifty-three children have active disease. Twenty-two children with JIA had a disease duration of ≤3 months, and 44 had >3 months.

### Dietary PUFA intake

We did not find significant differences in energy intake from dietary fats and polyunsaturated fatty acids between JIA patients and the healthy control (Table 2). There were no significant differences also in the PUFA intake measured grams pro day and grams pro MJ between categorized groups (data not shown). The diet met the recommended total energy and ALA intake in both groups. LA provided the close-to-recommended (for children) 4% energy (according to the European Food Safety Authority (EFSA) 2010, adopted by the National Food and Nutrition Institute, Warsaw, Poland) [23], and the n6/n3 ratio, circa 5.5% in both groups, did not indicate an excessive n6 PUFA level in the diet. However, the n3 long-chain PUFA intake was very low in the studied children and provided less than 0.03% of energy (when measured grams pro day: 0.031 and 0.066 in JIA and control groups, respectively, data not shown in Table 2). The median of EPA + DHA intake comprised only 12 and 26% of recommendation for children (0.25 g/day) [23].

**Table 2:** Dietary fat intake in children with JIA and control group

Nutritional intake	JIA group, *n* = 66	Control group, *n* = 42	*p* value
Dietary fat (E%)	26.85 (16.56–47.9)	29.95 (18.4–43.77)	0.88
n-6 PUFA (E%)	3.04 (1.30–4.88)	3.05 (1.30–9.95)	0.25
LA (E%)	2.97 (1.27–4.82)	3.44 (1.77–9.94)	0.60
AA (E%)	0.007 (0.001–0.03)	0.007 (0.001–0.03)	0.09
n-3 PUFA (E%)	0.56 (0.32–1.38)	0.54 (0.27–2.67)	0.50
ALA (%E)	0.48 (0.29–1.13)	0.46 (0.24–1.96)	0.32
EPA (%E)	0.006 (0.00–0.19)	0.007 (0.00–0.60)	0.31
DHA (%E)	0.02 (0.004–0.56)	0.02 (0.002–0.79)	0.69

Values given as median (range)

*AA* arachidonic acid, *ALA* α-linolenic acid, *DHA* docosahexaenoic acid, *EPA* eicosapentaenoic acid, *FA* fatty acids, *JIA* juvenile idiopathic arthritis, *LA* linoleic acid, *PUFA* polyunsaturated fatty acids, *%E* proportion of total energy intake

### Serum PUFA profile

**Fig. 1:**
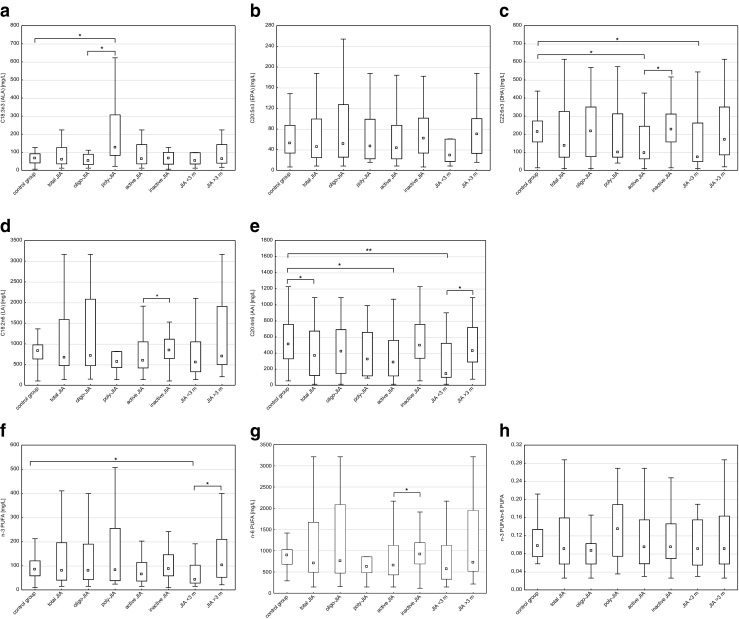
Serum PUFA level in children with JIA and in the control group, in milligrams per liter. **a** C18:3n3 (ALA). **b** C20:5n3 (EPA). **c** C22:6n3 (DHA). **d** C18:2n6 (LA). **e** C20:4n6 (AA). **f** n-3 PUFA. **g** n-6 PUFA. **h** n-3 PUFA/n-6 PUFA. Abbreviations: *AA* arachidonic acid; *ALA* α-linolenic acid, *DHA* docosahexaenoic acid, *EPA* eicosapentaenoic acid, *FA* fatty acids, *JIA* juvenile idiopathic arthritis, *LA* linoleic acid, *PUFA* polyunsaturated fatty acids, *n3* LA + AA, *n6* ALA + EPA + DHA. *p < 0.05, **p < 0.005

Results of serum PUFA level in children with JIA and in the control group are summarized in Fig. 1.

Our study showed that levels of total n-6 PUFA and LA were significantly higher in inactive JIA patients compared to active JIA patients (*p* = 0.018, *p* = 0.024, respectively). JIA patients, particularly active JIA patients and with short-lasting disease, had significantly lower levels of AA than the healthy control (*p* = 0.003, *p* = 0.012, *p* = 0.002, respectively). Furthermore, AA levels and total n-3 PUFA were significantly lower in JIA patients with short-lasting than with long-lasting disease (*p* = 0.009, *p* = 0.040, respectively). We also found that ALA level in serum of patients with poly-JIA was significantly higher than in the oligo-JIA group and in healthy controls (*p* = 0.042, *p* = 0.022, respectively). Levels of DHA were significantly lower in active and with short-lasting disease JIA patients in comparison to the healthy control group (*p* = 0.015, *p* = 0.024, respectively). DHA levels were also lower in active patients than in inactive patients (*p* = 0.028).

### Cytokines

Table 3 summarizes the serum concentration of IL-6, IL-17, and IL-10 in children with JIA and the control group. No significant differences were found in IL-6 concentration in patients with JIA compared to the control group, irrespective of the disease subtype, activity, or duration. We found significantly higher serum IL-10 levels in JIA patients, especially with active disease, in oligo- and poly-JIA, as well as in groups of patients with short-lasting and long-lasting disease in comparison to the healthy control group (*p* = 0.008, *p* = 0.008, *p* = 0.047, *p* = 0.018, *p* = 0.036, *p* = 0.020, respectively).

**Table 3:** Interleukin level in children with JIA and in the control group

Interleukin	Control group, *n* = 42	Total JIA, *n* = 66	Oligo-JIA, *n* = 38	Poly-JIA, *n* = 18	Active JIA, *n* = 53	Inactive JIA, *n* = 13	JIA duration ≤3 months, *n* = 22	JIA duration >3 months, *n* = 44
IL-6	10.62 (0.16–74.20)	10.61 (0.16–62.78)	22.88 (0.16–62.78)	15.00 (11.52–60.51)	16.86 (0.16–62.78)	5.38 (1.40–8.33)	12.88 (1.82–62.78)	5.38 (016–60.51)
IL-17	11.85 (0.20–32.79)	12.19 (1.24–62.90)	11.22 (1.24–54.91)	16.57 (1.71–62.90)	13.62 (1.57–62.90)	10.57 (1.24–50.29)	21.84 (1.57–62.90)	11.22 (1.24 54.91)
IL-10	5.06 (0.06–25.47)	10.26 (1.91–99.96)*	17.38 (1.91–99.96)**	32.58 (12.28–98.88)**	19.36 (1.91–99.96)*	10.26 (3.42–27.57)	12.19 (1.91–99.96)**	27.57 (2.28–98.88)**

All values median (range) are expressed in pg/mL

*IL-6* interleukin 6, *IL-17* interleukin 17, *IL-10* interleukin 10, *JIA* juvenile idiopathic arthritis

*p < 0.01 vs. “Control group”; *p <* 0.05 vs. control group

### Correlation between dietary intake of PUFAs and serum PUFA level

We did not find a significant correlation between dietary intake and serum level of PUFA between JIA patients and the healthy control (data not shown).

### Association analysis of serum PUFAs and clinical and laboratory JIA activity markers

We found that the n-3 and n-6 PUFAs negatively correlated with classical markers of inflammation CRP and ESR (*p* < 0.05) and positively correlated with platelet count (*p* < 0.05) (Table 4).

**Table 4:** Relationship between serum PUFAs level, inflammatory markers, and clinical findings of JIA

		Serum PUFA level						
		Σn-6 PUFA	C18:2n6 (LA)	C20:4n6 (AA)	Σn-3 PUFA	C18:3n3 (ALA)	C20:5n3 (EPA)	C22:6n3 (DHA)
Inflammatory parameters								
ESR	JIA group	**−0.427 (0.002)**	**−0.418 (0.002)**	**−0.403 (0.003)**	**−0.399 (0.003)**	−0.242 (0.084)	**−0.374 (0.006)**	**−0.431 (0.001)**
CRP	JIA group	**−0.494 (0.0002)**	**−0.471 (0.0005)**	**−0.612 (0.000002)**	**−0.517 (0.0001)**	**−0.387 (0.005)**	**−0.501** **(0.0002)**	**−0.493 (0.0002)**
WBC count	JIA group	0.105 (0.459)	0.116 (0.414)	−0.039 (0.785)	0.008 (0.952)	0.050 (0.726)	0.024 (0.866)	0.041 (0.773)
PLT count	JIA group	**0.381 (0.005)**	**0.360 (0.009)**	**0.383 (0.005)**	**0.316 (0.022)**	**0.330 (0.017)**	**0.312** **(0.024)**	0.255 (0.069)
Clinical findings								
Active joints count	JIA group	**−0.319 (0.021)**	**−0.292 (0.036)**	**−0.404 (0.003)**	−0.228 (0.105)	−0.008 (0.953)	−0.210 (0.135)	**−0.341 (0.013)**
Disease duration	JIA group	0.246 (0.079)	0.226 (0.107)	**0.372 (0.007)**	**0.341 (0.013)**	0.149 (0.290)	**0.299** **(0.031)**	0.268 (0.055)

Values given as Rho (*p*). Bold values are present statistical significant values

*AA* arachidonic acid, *ALA* α-linolenic acid, *CRP* C-reactive protein, *DHA* docosahexaenoic acid, *EPA* eicosapentaenoic acid, *ESR* erythrocyte sedimentation rate, *FA* fatty acids, *JIA* juvenile idiopathic arthritis, *LA* linoleic acid, *PUFA* polyunsaturated fatty acids, *PLT* platelet, *WBC* white blood cells, *Σn3* LA + AA, *Σn6* ALA + EPA + DHA

Our study revealed that in JIA patients, the total n-6 PUFA, LA, AA, and DHA levels negatively correlated with the number of active joints, and the AA, total n-3 PUFA, and EPA levels positively correlated with duration of the disease (Table 4). No correlations of PUFA and cytokines were found (data not shown).

## Discussion

To our knowledge, this is the first study that explores the serum profile of PUFAs in JIA patients without omega-3 fatty acid supplementation. We aimed to investigate whether the PUFA metabolic pathway is affected and to what extent in patients with JIA.

### PUFA dietary intake

In our study, dietary intake of PUFA by the JIA patients was similar to that of the control group. Our findings are consistent with previous reports which showed no differences in nutrient intake between sero-negative poly-JIA and oligo-JIA subtypes, or between the children with active and inactive disease [24]; no association between fat intake and disease activity was found either [25]. Such results are not surprising as no intervention in diet nor n-3/n-6 PUFA supplementation was designed in this study, and both JIA and healthy children continued their regular daily diet.

We found no correlations between dietary and serum PUFA. A lack of association between dietary intake and blood fatty acid concentration in children and adults had been reported earlier [16, 26]; to date, other authors found inconsistent correlations in diet supply and serum levels, in n-3 PUFA (but not n-6) [27, 28], or the opposite [17]. Interestingly, a recent systematic review demonstrated that LA and AA intake is reflected by plasma fatty acids, but LA intake had not consistently been shown to increase the conversion of this fatty acid to AA, nor had AA consistently been shown to affect EPA or DHA concentration [29].

Among serum PUFA levels, especially DHA seems to be most sensitive to dietary intake and supplementation [30]. However, it has been shown that adequate dietary ALA intake may ensure ample DHA level in blood by effective conversion of ALA in liver [31]. We have found significantly lower serum DHA level in patients with early-stage and active JIA when compared to the control group despite a lack of differences in PUFA intake between studied groups of children, as well as intake of nutrients (dietary fiber, sugars, data not shown) which have been proven to affect plasma DHA levels in children [32]. This may suggest the impeded endogenic synthesis of DHA in active and short-lasting disease [30, 32]. This result implies a need for more information from larger, dose-response studies with meticulous dietary control, especially in young patients.

### Serum PUFA level

The theoretical background of the “linoleic acid pro-inflammatory paradigm” is based on an enhanced synthesis of pro-inflammatory eicosanoids derived from AA and diminished synthesis of anti-inflammatory eicosanoids from EPA and DHA, observed mostly in in vitro and in multiple animal experiments [7]. On the other hand, the increasing number of evidences from human studies suggests that n-6 PUFAs also provide some anti-inflammatory activity, similarly to n-3 PUFA [29, 33]. We found lower serum levels of AA and DHA in JIA patients, particularly in active patients and with short-lasting disease, than in the healthy control group, and higher n-6 LA level when comparing an inactive vs active disease period. A decreased concentration of AA and increased concentration of LA in phosphatidylcholine of the erythrocyte membranes in children with JIA, and during the active period of JIA, was also reported in another study [12]. Significantly reduced n-3 and n-6 PUFA serum levels were noted in adult SLE patients [34]. Keeping in mind the results of our study, we assume that during an active period in the beginning of a disease the metabolic disturbances occur. They could be related to desaturation and elongation defects or increased beta-oxidation of lipids [34]. The outcome is an insufficient concentration of n-3 and n-6 metabolites and predomination of a pro-inflammatory response. Another explanation might be the recent data of a crucial role of “fatty acid metabolism-immunity nexus” (FAMIN) protein as the central regulator of endogenous fatty acid synthesis and their mitochondrial oxidation in macrophages. A mutation within the FAMIN gene was found in children with JIA [35, 36]. A lack of significant differences in the concentration of LA and ALA between the JIA group and healthy children (except of higher ALA level in poly-JIA) may additionally suggest a role of their metabolites in the mechanism of JIA.

Our observation of the altered blood composition of n-6 and n-3 PUFA is not, as we believe, associated with an inadequate provision in the diet: first, in our study, the nutritional status of children of both groups did not differ on a statistically significant level; second, our study revealed that dietary PUFA intake is not associated with serum PUFA. Noteworthily, Cleary et al. [37] reported that nutritional impairment in JIA is not an effect from an inadequate food intake, but is associated with the disease subtype.

Nonetheless, there are too few evidences to define whether an altered serum PUFA profile could be modified and how dietary fats may affect disease activity in JIA patients.

### Cytokines

We found an increased IL-10 serum level in patients with active oligo- and poly-JIA, regardless of the disease time span, in comparison to the healthy control group. Other authors had published that IL-10 was significantly elevated in specific subtypes of JIA, e.g., poly-JIA and in sJIA [38, 39]. An increase of IL-10 in the active stage of the disease suggests that the anti-inflammatory effects may be reduced during systemic flares [38, 40], or alternatively, that remarkable upregulation of genes associated with IL-10 signaling in recent onset occurs in untreated JIA patients [41].

No significant differences in the concentration of IL-6 in patients with JIA compared to the control group, irrespective of the disease subtype, activity, or duration time, were revealed in our study, which is in agreement with previous data [2, 24]. To date, IL-6 is mostly attributed to the pathogenesis of sJIA and elevated serum level is observed in the active disease period [3, 38]. In our study, however, patients with sJIA accounted only for 4.5%, as the majority represented oligo- and poly-JIA types of the disease. van den Ham et al. [42] showed that oligo- and poly-JIA have similar biomarker profiles, both in plasma and synovial fluid.

In this study, the serum PUFA level did not correlate with any of the examined cytokines. Experimental studies revealed that dietary n-3 PUFA and n-3 PUFA added to cells in vitro did not affect IL-10 nor IL-6 secretion [43, 44], and interventional studies in a group of healthy adults stated that habitual intake of LA and ALA is not related to plasma IL-6 levels [45]. Ferrucci et al. [46] in a comprehensive study had examined the relationship between PUFA concentration in plasma and inflammatory marker levels in healthy adults and found that total n-3 fatty acids were independently associated with decreased level of pro-inflammatory markers (IL-6, IL-1ra, TNF-α, C-reactive protein), as well as increased level of anti-inflammatory markers (soluble IL-6r, IL-10, TGF-β). Altogether, the relationship between PUFA and cytokine production is inconclusive.

### Disease activity

The most interesting findings in our study concerned correlations between n-6 and n-3 PUFA levels and clinical activity of disease as well as registered laboratory parameters.

In the present study, serum n-6, LA, AA, and DHA levels were inversely associated with number of active joints. Górska et al. [12] had observed a decreased concentration of AA in phosphatidylcholine in the erythrocyte membranes during the active period of JIA. To our knowledge, the only study describing an impact of n-3 PUFA supplementation for disease activity in patients with JIA was published by Gheita et al. [11] who demonstrated that supplementation of n-3 PUFA (2 g per day over 12 weeks) improved clinical outcome (by decreasing the amount of active joints, number of swollen joints, JADAS-27, CHAQ) [11]. Obviously, these findings need a critical discussion, as the study covered a relatively small group of patients, and, above all, during the intervention all the patients were treated with DMARDs: methotrexate, hydroxychloroquine, leflunomide, sulfasalazine, or steroids, and there was also no control group or placebo. Thus, it is unsolved whether the therapeutic effect was in fact a result of supplementation or traditional medication.

The relationship of PUFAs with the duration of rheumatoid arthritis was recently shown by Proudman et al. [47], who in patients with recent RA onset (<12 months) found that a rate of first remission was significantly higher in the group consuming fish oil (providing 5.5 g/day EPA + DHA.). The positive relationship between serum AA, n-3, EPA level, and disease duration in our study remains consistent with these observations and supports the suggestion that the dietary interventions would be justified in the early stages of disease.

We found a strong negative correlation between serum n-6 and n-3 PUFA and the markers of inflammation (ESR, CRP). The negative relationship between serum n-3 PUFA compositions and correlating CRP level in adults has been reported by other authors [48, 49]. Pirschon et al. [45] in a large population found an inverse relationship between an intake of n-3 PUFA and CRP levels, as well as a strong inverse association between high n-6 intake and soluble tumor necrosis factor receptors 1 and 2. The authors concluded that the n-3 fatty acids in combination with n-6 fatty acids cause minute decrease of pro-inflammatory cytokine concentration [45]. Additionally, in our study we observed that elevated serum total n-6, LA, AA, total n-3, ALA, and EPA levels were associated with an increased platelet count. Interestingly, platelets activated by pro-inflammatory cytokines (e.g., TNF-α, IL-6) play an important role in the pathogenesis of joint inflammation [50, 51]. Summing up, our data shows that both n-6 and n-3 PUFA may be engaged in decreasing inflammation during JIA.

### Limitations

Although our multicenter study involved altogether a large group of children with JIA (*n* = 66), the individual subgroups are not numerous. The study was designed as a single sight on assessment; there was no dietary intervention either. Nevertheless, dividing patients into subgroups allowed us to present a complete picture of interactions. Using these results, patients for whom an intervention study could be warranted have been identified.

## Conclusion

To our knowledge, this is the first study that simultaneously investigated an association between dietary intake of n-3 and n-6 PUFA, serum PUFA profile, and inflammatory markers among children with JIA. The combination of reduced levels of AA and DHA in the active phase of poly-JIA with the increased concentration of IL-10, the inverse relationship of the two trails (n-6 and n-3), PUFA with classic markers of inflammation (CRP, ESR), and positive correlation with platelet count had indicated participation of PUFAs in the early stages of poly-JIA pathogenesis. Our results may support an identification of the target group of patients with JIA to whom PUFA supplementation in addition to regular daily diet might be recommended in order to achieve a positive immunomodulating effect. Further studies and evidences are required, and strongly advised, prior to incorporation of such therapy in JIA patients.
